# Supplementary material for: The Predicted Functional Compartmentation of Rice Terpenoid Metabolism by Trans-Prenyltransferase Structural Analysis, Expression and Localization
Source: Int J Mol Sci. 2020 Nov 25;21(23):8927. doi: 10.3390/ijms21238927 (PMC7728057; doi:10.3390/ijms21238927)
Supplement: Supplementary file 1 [file ijms-21-08927-s001.pdf]

Table S1. Primer list for expression profiling of PT genes

Genes	Primer sequences	Gene ID
OsGPS	5'-CCCGAAGCTGCTCGGCGTCGACAAGG-3'<br>5'-GAGCCATCAATTAATGCTGCCTGTAG-3'	Os01g14630
OsGGPS1	5'-AGGCTCCGAGGGCCTAGTCGCCGGCCAGGTTGTTG-3'<br>5'-ATTGCCCCAATAACCACTGATGCCTC-3'	Os07g39270
OsGRP	5'-ACGGCCGCACCATCGGCGTCCTGTACCAGCTCGTC-3'<br>5'-TGCGCTTTGAGCTCCTCTACGATGCC-3'	Os02g44780
OsSPS1	5'-TCGCATATGAATATGGTCGAAACCTGGGTTTAGCC-3'<br>5'-ATAGGTGCGGTAATGATTCCATGACG-3'	Os06g46450
OsSPS2	5'-CCAGTTCAGAAGTACCCTTGAGAACGTG-3'<br>5'-CAAGATGAACTTCTTTGGGAGCTACTAAC-3'	Os05g50550
OsSPS3	5'-ACTGCAATAGAGCTAGTTCATAGAAGTGG-3'<br>5'-GGAGCTACTGTAAGCTCACAAAGGAGG-3'	Os12g17320
OsSPS4	5'-ACCCCTCAAATGTAGACGCAGCCCTT-3'<br>5'-CTTGCTCTCAGGAAGAGCATCGATTGCG-3'	Os08g09370
OsFPS1	5'-GATCTGTATAAAGAACTTAATCTGGAGGCCG-3'<br>5'-AACATGAAACAGCTTCTCCATGA-3'	Os01g50760
OsFPS2	5'-AGAGAACTAAACCTAGAGGCGGTC-3'<br>5'-CTAGAGAAGAAACATGCAACAATGGACAGC-3'	Os05g46580
OsFPS3	5'-AACGAACTTCATCTCCAGCGGGTG-3'<br>5'-GCCAAGAACCAGAATAATCCACGAGATGCC-3'	Os01g50050
OsFPS4	5'-ACAGGGAGCTTGATCTTCAGGAC-3'<br>5'-ATCTTCTTCAGAAACGACTTCAAAATATCCCG-3'	Os04g56230
OsFPS5	5'-GCTGACAACAATCAAATAGAAGTACTACATAGG-3'<br>5'-GCATGATCCTTCTGAGCTTCGATAG-3'	Os04g56210
OsTPS19	5'-GCATATACATTTTCGAAGTATCATCAGGGA-3'<br>5'-GATTTATGTACAAAAGTGAACTTATTTTAAGAT-3'	Os04g27190
OsPSY1	5'-GGGAAGATGATGAGCAGGTTA-3'<br>5'-GCATTTTCCCTATACATGCT-3'	Os06g51290
OsGA2ox3	5'-ACGTCGGCGACTCGTTGCAGGTTC-3'<br>5'-CAGCTGTGGCAATGGTGCAATCCTC-3'	Os01g55240
OsUbi5	5'-GAAGTAAGGAAGGAGGAGGA-3'<br>5'-AAGGTGTTCAGTTCCAAGG-3'	Os01g22490
OsEF1a	5'-CTGCAGACATGCAAACCACCATTTGAAC-3'<br>5'-AGGCAAACGGTGGCTGTTGGCGTCATC-3'	Os03g08050

Table S2. Primers for cloning into pDONR221-Kz-*Nde* I-sGFP

Genes	Primer sequences	Product sizes
OsGPS	5'-GTACAAAAAAGCAGGCTCATATGACTTTTCTCTATTTAAACACCATCCCCGACTC-3'<br>5'-TACAAGAAAGCTGGGTCATATGATGCTGCCTGTAGGCGATGAACCG-3'	1290 bp
OsGGPS1	5'-GTACAAAAAAGCATATGGCTGCCTTCCCCCCGCTCGCCGCCTCCCG-3'<br>5'-AAGCTGGGTCATATGGTTCTGCCGATAGGCAATATAATTGGCCAGGTGCAGAAGTGGT-3'	1127 bp
OsGRP	5'-GGGGACAAGTTTGCATATGGCTCTCTCCTCCTTCTCCATGTCCCTCCCCTTCG-3'<br>5'-TTACAAGAAACATATGCGGCGTTGTAGCTGCATCCTGCAGCTCGAATCCCCTC-3'	1058 bp
OsSPS1	5'-GTAGTTTGCGGCCGCGTCGTGGCGGTGGGCTCTCGCGCGGCGCGTC-3'<br>5'-TCTTAAGCTGCGGCCGCTTTTGTTCTAGTGATGACTCTCTCTGTGATG-3'	1320 bp
OsSPS2	5'-AAAAAAGCAGGCTCATATGTTGTCTGTGAGCTGCCCGAGAGTGT-3'<br>5'-TACAAGAAAGCTGGGTCATATGGTCAATCCTCTGAAGATTATATTTCACCACG-3'	1247 bp
OsSPS3	5'-GCAGGCTCATATGATGGCGGCGCCGTCCTCCCTCGCG-3'<br>5'-AGCTGGGTCATATGATCAATCCTTTCTAGATTATATTTCAC-3'	1269 bp
OsSPS4	5'-GGACGGGCTCATATGCTACTGCCGAGGCGTCTTCCTCTTCTTGCTG-3'<br>5'-AAGCTGGGTCATATGCTTTGTTCTCTTGATGAGCTTTTCTGTAAG-3'	1224 bp
OsFPS1	5'-CGCGTAGTGATCATATGGCGGCGGCGGTGGTGGCGAAC-3'<br>5'-TCTTAAGCTCATATGCTTCTGCCTCTTGTAGATCTTGTGCAGGA-3'	1088 bp
OsFPS2	5'-CGCGTAGTGATCATATGGCGGCGGCGAATGGGAG-3'<br>5'-GGGCGACGCTCATATGCTTCTGTCTCTTGTAGATCTTGTG-3'	1095 bp
OsFPS3	5'-CGCGTAGTGATCATATGGCCGCCGCGGGTG-3'<br>5'-GCGGTCTTAAGCTCATATGCTTGCTCCTCTTGTAGATCTTGTG-3'	1101 bp
OsFPS4	5'-ATACCGCGTAGTGATCATATGGGGGCGCTGGCGGCGGAG-3'<br>5'-TCTTAAGCTCATATGCTTCTTCCTCCTGTAAATCTTCTTCAGAAACGAC-3'	1095 bp
OsFPS5	5'-AAAAAAGCAGGCTCATATGTCCATGCAGGAGATGCTCCGCATTG-3'<br>5'-TACAAGAAAGCTGGGTCATATGCTTCTTCCTTTGATGAATTTTCTTCAGAAATG-3'	1214 bp

Table S3. The list of plant trans-prenyltransferases analyzed in phylogenetic tree analysis.

Groups	Plant group	Name	Plant Species	Accession No.	Size (aa)
I. GPS/GGPS/GFPS	Dicotyledon /Angiosperm	Am AAS82860	*Antirrhinum majus*	AAS82860	372
		Am AAS82859	*A. majus*	AAS82859	297
		At NP195399	*Arabidopsis thaliana*	NP_195399	371
		At ABE65825	*A. thaliana*	ABE65825	347
		At AAK00407	*A. thaliana*	AAK00407	326
		AT3G32040	*A. thaliana*	AT3G32040	360
		AT3G29430	*A. thaliana*	AT3G29430	357
		AT3G14510	*A. thaliana*	AT3G14510	284
		AT3G14550	*A. thaliana*	AT3G14550	360
		AT3G14530	*A. thaliana*	AT3G14530	360
		Cr AGL91645	*Catharanthus roseus*	AGL91645	383
		Cr AGL91646	*C. roseus*	AGL91646	299
		Cb AAS82870	*Clarkia breweri*	AAS82870	300
		Ca ABW06960	*Corylus avellana*	ABW06960	373
		Hb BAF98303	*Hevea brasiliensis*	BAF98303	306
		Hb BAB60678	*H. brasiliensis*	BAB60678	370
		Hb BAF98300	*H. brasiliensis*	BAF98300	306
		Hl ACQ90682	*Humulus lupulus*	ACQ90682	369
		Hl ACQ90681	*H. lupulus*	ACQ90681	279
		Js AIY24421	*Jasminum sambac*	AIY24421	360
		Mi AFJ52722	*Mangifera indica*	AFJ52722	328
		Ms ADG01841	*Medicago sativa*	ADG01841	370
		Mp AAF08793	*Mentha x piperita*	AAF08793	377
		Mp AAF08792	*Mentha x piperita*	AAF08792	313
		Nt AFB35651	*Nicotiana tabacum*	AFB35651	
		Nt NP001312106	*N. tabacum*	NP_001312106	
		Nt ADD49735	*N.tabacum*	ADD49735	365
		Sm AEZ55680	*Salvia miltiorrhiza*	AEZ55680	331
		Sm AEZ55681	*S. miltiorrhiza*	AEZ55681	344
		Sm ACR19637	*S. miltiorrhiza*	ACR19637	346
		Sm AEZ55682	*S. miltiorrhiza*	AEZ55682	346
		Sm AEZ55683	*S. miltiorrhiza*	AEZ55683	379
		Sm AEZ55678	*S. miltiorrhiza*	AEZ55678	314
		Sm AEZ55679	*S. miltiorrhiza*	AEZ55679	290
		Sa CAA67330	*Sinapis alba*	CAA67330	366
		Sl ABB82554	*Solanum lycopersicum*	ABB82554	365
		Sl ABB82555	*S. lycopersicum*	ABB82555	363
		Vv CAO38946	*Vitis vinifera*	CAO38946	298
		Vv CAO64763	*V. vinifera*	CAO64763	341
	Gymosperm	Ag AAN01133	*Abies grandis*	AAN01133	383
		Ag AAN01134	*A. grandis*	AAN01134	381
		Ag AAN01135	*A. grandis*	AAN01135	387
		Eu ACO59905	*Elaeagnus umbellate*	ACO59905	365
		Gb AAQ72786	*Ginkgo biloba*	AAQ72786	391
		Jc ADD82422	*Jatropha curcas*	ADD82422	370
		Pa ACA21458	*Picea abies*	ACA21458	386
		Pa ACZ57571	*P. abies*	ACZ57571	383
		Pm AGU43761	*Pinus massoniana*	AGU43761	380
		Tm AAS67008	*Taxus x media*	AAS67008	393
	Monocotyledon /Angiosperm	Hv BAJ94484	*Hordeum vulgare*	BAJ94484	358
		Hv BAJ96219	*H. vulgare*	BAJ96219	342
		Os BAT02334	*Oryza sativa*	BAT02334	366
		Os XP015626863	*O. sativa*	XP_015626863	342
		Os BAS71331	*O. sativa*	BAS71331	416
		Sb XP021311468	*Sorghum bicolor*	XP_021311468	366
		Sb XP002463084	*S. bicolor*	XP_002463084	371
		Sb XP002452775	*S. bicolor*	XP_002452775	342

		Ta SPT17215	*Triticum aestivum*	SPT17215	356
		Ta BT009222	*T. aestivum*	BT009222	345
		Zm ABQ85648	*Zea mays*	ABQ85648	363
		Zm NP001183930	*Z. mays*	NP_001183930	367
		Zm XP008678927	*Z. mays*	XP_008678927	342
**II. SPS/PPS**	**Dicotyledon /Angiosperm**	At ABF58968	*Arabidopsis thaliana*	ABF58968	406
		At ABI54337	*A. thaliana*	ABI54337	417
		At AAW39025	*A. thaliana*	AAW39025	422
		Hb BAF98298	*H.brasiliensis*	BAF98298	330
		Hb ABD92707	*H.brasiliensis*	ABD92707	418
		Mi AFJ52721	*M. indica*	AFJ52721	421
		Nt AHL84161	*N. tabacum*	AHL84161	415
		Sm AEZ55677	*S. miltiorrhiza*	AEZ55677	424
		Sl ABB88703	*S. lycopersicum*	ABB88703	415
		Sl ABI63627	*S. lycopersicum*	ABI63627	398
		Cu AAN86061	*Citrus unshiu*	AAN86061	426
		Vv CAO17862	*V. vinifera*	CAO17862	422
	**Gymosperm**	Pa ACA21459	*P. abies*	ACA21459	427
		Qr CAC20852	*Quercus robur*	CAC20852	416
	**Monocotyledon /Angiosperm**	Hv BAK05302	*H. vulgare*	BAK05302	424
		Hv BAK00672	*Hordeum vulgare*	BAK00672	394
		Os BAS99121	*O. sativa*	BAS99121	430
		Os Q75HZ9	*O. sativa*	Q75HZ9	403
		Os XP015620200	*O. sativa*	XP_015620200	414
		Os XP015648843	*O. sativa*	XP_015648843	412
		Sb XP002438852	*S. bicolor*	XP_002438852	431
		Sb XP021321875	*S. bicolor*	XP_021321875	489
		Sb XP021321027	*S. bicolor*	XP_021321027	395
		Ta AK334813	*T. aestivum*	AK334813	426
		Ta BT009280	*T. aestivum*	BT009280	402
		Ta AK331526	*T. aestivum*	AK331526	402
		Zm XP008643450	*Z. mays*	XP_008643450	428
		Zm NP001347618	*Z. mays*	NP_001347618	424
	**Dicotyledon /Angiosperm**	At AAL34286	*A. thaliana*	AAL34286	385
		At CAB80990	*A. thaliana*	CAB80990	342
		At AAB07248	*A. thaliana*	AAB07248	342
		Aa ADJ67472	*Artemisia annua*	ADJ67472	343
		As AAP74719	*Artemisia spiciformis*	AAP74719	342
		Br XP009128999	*Brassica rapa*	XP_009128999	387
		Ca CAA59170	*Capsicum annuum*	CAA59170	347
		Cr ADO95193	*C. roseus*	ADO95193	345
		Cl AFW98433	*Chrysanthemum lavandulifolium*	AFW98433	344
		Cs XP006474825	*Citrus sinensis*	XP_006474825	342
		Cm XP008463060	*Cucumis melo*	XP_008463060	342
		Hb AAM98379	*H. brasiliensis*	AAM98379	342
		Hl BAB40665	*H. lupulus*	BAB40665	342
		La AGQ04160	*Lavandula angustifolia*	AGQ04160	349
		Md AAM08927	*Malus x domestica*	AAM08927	342
		Mi AFJ52720	*M. indica*	AFJ52720	342
		Ms ADC32809	*M. sativa*	ADC32809	342
		Mt XP003594327	*Medicago truncatula*	XP_003594327	342
		Mp AAK63847	*Mentha x piperita*	AAK63847	349
		Nt AHM22931	*N. tabacum*	AHM22931	341
		Pg AAY87903	*Panax ginseng*	AAY87903	342
		Pn AAY53905	*Panax notoginseng*	AAY53905	343
		Pq ADJ68004	*Panax quinquefolius*	ADJ68004	342
		Sm ABV08819	*S. miltiorrhiza*	ABV08819	349
		Sl NP001234068	*S. lycopersicum*	NP_001234068	342

		Vv AAX76910	*V. vinifera*	AAX76910	341
**III. FPS**		Gb AAR27053	*Ginkgo biloba*	AAR27053	390
	**Gymosperm**	Pa ACA21460	*P. abies*	ACA21460	347
		Tm AAS19931	*Taxus media*	AAS19931	351
		Ao ADV03674	*Alisma orientale*	ADV03674	343
		Ap ADR83704	*Alisma plantago-aquatica*	ADR83704	343
		Hc AER12202	*Hedychium coccineum*	AER12202	356
		Cg AFP19446	*Cymbidium goeringii*	AFP19446	348
		Do AFX68799	*Dendrobium officinale*	AFX68799	348
		Hv BAJ84778	*H. vulgare*	BAJ84778	355
		Hv BAJ90047	*H. vulgare*	BAJ90047	352
		Hv BAJ87514	*H. vulgare*	BAJ87514	408
		Ll ADZ57167	*Lilium longiflorum*	ADZ57167	351
		Ol AHA51120	*Ornithogalum longebracteatum*	AHA51120	347
		Os BAS73911	*O. sativa*	BAS73911	353
	**Monocotyledon /Angiosperm**	Os BAS95151	*O. sativa*	BAS95151	355
		Os BAS73843	*O. sativa*	BAS73843	307
		Os MT793647	*O. sativa*	MT793647	354
		Os BAS91413	*O. sativa*	BAS91413	392
		Pd XP008792798	*Phoenix dactylifera*	XP_008792798	350
		Sb XP002456217	*S. bicolor*	XP_002456217	356
		Sb XP002441458	*S. bicolor*	XP_002441458	353
		Sb XP021319712	*S. bicolor*	XP_021319712	405
		Ta AFV51836	*T. aestivum*	AFV51836	354
		Ta AGC11812	*T. aestivum*	AGC11812	351
		Ta AK330255	*T. aestivum*	AK330255	410
		Zm ACR35378	*Z. mays*	ACR35378	355
		Zm ACF88176	*Z. mays*	ACF88176	350

Table S4. The list of plant trans-prenyltransferases analyzed in multiple sequence alignment.

Groups	Protein ID	Plant Species	Accession No.	Subgroups	Size (aa)
Group-I	AT4G36810_A	*Arabidopsis thaliana*	AT4G36810	A	371
	Mp AAF08793_A	*Mentha x piperita*	AAF08793	A	377
	OsGGPS1_A	*Oryza sativa*	BAT02334	C	366
	Zm NP001183930_A	*Zea mays*	NP001183930	C	367
	Sb XP002463084_A	*Sorghum bicolor*	XP002463084	C	371
	Hv BAJ94484_A	*Hordeum vulgare*	BAJ94484	C	358
	Ta SPT17215_A	*Triticum aestivum*	SPT17215	C	356
	AT3G14550_B	*A. thaliana*	AT3G14550	B	360
	AT3G29430_B	*A. thaliana*	AT3G29430	B	357
	AT1G49530_C	*A. thaliana*	AT1G49530	E	336
	AT2G18640_C	*A. thaliana*	AT2G18640	E	372
	OsGPS_C	*O. sativa*	BAS71331	E	416
	Zm ABQ85648_C	*Zea mays*	ABQ85648	E	363
	Sb XP021311468_C	*S. bicolor*	XP021311468	E	366
	AgAAN01133_D	*Abies grandis*	AAN01133	D	383
	PaACA21458_D	*Picea abies*	ACA21458	D	386
	Mp AAF08792_E	*M. x piperita*	AAF08792	G	313
	OsGRP_F	*O. sativa*	XP015626863	F	342
	Zm XP008678927_F	*Zea mays*	XP008678927	F	342
	Sb XP002452775_F	*S. bicolor*	XP002452775	F	342
	Hv BAJ96219_F	*H. vulgare*	BAJ96219	F	342
	Ta BT009222_F	*T. aestivum*	BT009222	F	345
	AT4G38460_F	*A. thaliana*	AT4G38460	F	326
Group-II	OsSPS3_A	*O. sativa*	XP015620200	A	414
	Sb XP021321875_A	*S. bicolor*	XP021321875	A	489
	OsSPS2_A	*O. sativa*	Q75HZ9	A	403
	Hv BAK00672_A	*H. vulgare*	BAK00672	A	394
	Ta BT009280_A	*T. aestivum*	BT009280	A	402
	Zm NP001347618_A	*Zea mays*	NP001347618	A	424
	At ABF58968_A	*A. thaliana*	ABF58968	A	406
	At ABI54337_A	*A. thaliana*	ABI54337	A	417
	OsSPS4_B	*O. sativa*	XP015648843	B	412
	Sb XP021321027_B	*S. bicolor*	XP021321027	B	395
	At AAW39025_B	*A. thaliana*	AAW39025	B	422
	Zm XP008643450_B	*Zea mays*	XP008643450	B	428
	Sb XP002438852_B	*S. bicolor*	XP002438852	B	431
	Hv BAK05302_B	*H. vulgare*	BAK05302	B	424
	Ta AK334813_B	*T. aestivum*	AK334813	B	426
	OsSPS1_B	*O. sativa*	BAS99121	B	430
Group-III	Hv BAJ87514_A	*H. vulgare*	BAJ87514	A	408
	Ta AK330255_A	*T. aestivum*	AK330255	A	410
	OsFPS4_A	*O. sativa*	MT793647	A	354
	OsFPS5_A	*O. sativa*	BAS91413	A	392
	Sb XP021319712_A	*S. bicolor*	XP021319712	A	405
	OsFPS3_A	*O. sativa*	XP_015621770	A	356
	Hv BAJ84778_A	*H. vulgare*	BAJ84778	A	355
	Ta AFV51836_A	*T. aestivum*	AFV51836	A	354
	Sb XP002456217_A	*S. bicolor*	XP002456217	A	356
	OsFPS1_A	*O. sativa*	BAS73911	A	353
	Zm ACR35378_A	*Zea mays*	ACR35378	A	355
	Zm ACF88176_A	*Zea mays*	ACF88176	A	350
	Sb XP002441458_A	*S. bicolor*	XP002441458	A	353
	Hv BAJ90047_A	*H. vulgare*	BAJ90047	A	352
	Ta AGC11812_A	*T. aestivum*	AGC11812	A	351
	OsFPS2_A	*O. sativa*	BAS95151	A	355
	At AAL34286_B	*A. thaliana*	AAL34286	B	385
	At CAB80990_B	*A. thaliana*	CAB80990	B	342
	Nt AHM22931_B	*Nicotiana tabacum*	AHM22931	B	341
	Sm ABV08819_B	*S. bicolor*	ABV08819	B	349

Table S5. The differential amino acid residues of 'three floors' motifs in the families of Group-I, II and III.

Groups	Proteins	Floor1			Floor2			Floor3		
		Site 1	Site 2	Site 3	Site 1	Site 2	Site 3	Site 1	Site 2	Site 3
Group-I	AT4G36810_A	M	S	L	I	L	I	A	F	L
	Mp AAF08793_A	M	S	L	I	L	I	A	F	L
	OsGGPS1_A	M	S	L	V	L	I	A	F	L
	Zm NP001183930_A	M	S	L	V	L	I	A	F	L
	Sb XP002463084_A	M	S	L	V	L	I	A	F	L
	Hv BAJ94484_A	M	S	L	V	L	I	A	F	L
	Ta SPT17215_A	M	S	L	V	L	I	A	F	L
	AT3G14550_B	S	S	L	I	L	I	A	F	L
	AT3G29430_B	S	S	L	I	L	I	A	F	L
	AT1G49530_C	A	S	L	I	R	V	A	V	M
	AT2G18640_C	M	S	L	I	L	I	A	F	L
	OsGPS_C	M	S	V	I	H	S	A	F	L
	Zm ABQ85648_C	A	S	I	I	L	A	A	F	L
	Sb XP021311468_C	A	S	I	M	L	A	A	F	L
	AgAAN01133_D	M	S	V	I	L	I	A	F	L
	PaACA21458_D	M	S	V	I	L	T	A	F	I
	Mp AAF08792_E	A	A	M	V	V	G	A	F	I
	OsGRP_F	A	S	M	L	F	V	A	Y	L
	Zm XP008678927_F	A	S	M	L	F	V	A	Y	L
	Sb XP002452775_F	A	S	M	L	F	V	A	Y	L
	Hv BAJ96219_F	A	S	M	L	F	V	A	Y	L
	Ta BT009222_F	A	S	M	L	F	V	A	Y	L
	AT4G38460_F	A	S	M	V	F	V	A	F	I
Group-II	OsSPS3_A	T	A	F	I	F	I	A	-	I
	Sb XP021321875_A	T	A	F	I	F	I	A	-	I
	OsSPS2_A	T	A	F	I	F	I	A	-	I
	Hv BAK00672_A	T	A	F	I	F	I	A	-	I
	Ta BT009280_A	T	A	F	I	F	I	A	-	I
	Zm NP001347618_A	T	A	F	I	F	I	A	-	I
	At ABF58968_A	T	A	F	I	F	I	A	-	I
	At ABI54337_A	T	A	F	I	F	I	G	-	I
	OsSPS4_B	V	T	L	I	L	V	A	-	L
	Sb XP021321027_B	I	S	L	I	L	V	A	-	L
	At AAW39025_B	V	A	L	I	L	V	A	-	L
	Zm XP008643450_B	V	A	L	I	L	V	A	-	M
	Sb XP002438852_B	V	A	L	I	L	V	A	-	M
	Hv BAK05302_B	V	A	L	I	L	V	A	-	M
	Ta AK334813_B	V	A	L	I	L	V	A	-	M
	OsSPS1_B	V	A	L	I	L	V	A	-	M

	Hv BAJ87514_A	S	A	-	L	L	T	G	V	I
	Ta AK330255_A	S	A	-	L	L	T	G	V	I
	OsFPS4_A	S	A	-	L	L	T	G	V	I
	OsFPS5_A	C	A	-	F	L	T	G	I	I
	Sb XP021319712_A	C	A	-	F	L	T	G	I	I
	OsFPS3_A	Y	F	-	L	L	T	G	I	V
	Hv BAJ84778_A	Y	F	-	L	L	T	G	I	V
	Ta AFV51836_A	Y	F	-	L	L	T	G	I	V
	Sb XP002456217_A	Y	F	-	L	L	T	G	I	V
**Group-III**	OsFPS1_A	Y	F	-	L	L	T	G	I	V
	Zm ACR35378_A	Y	F	-	L	L	T	G	I	V
	Zm ACF88176_A	F	F	-	L	L	T	G	I	V
	Sb XP002441458_A	F	F	-	L	L	T	G	I	V
	Hv BAJ90047_A	F	F	-	L	L	T	G	I	V
	Ta AGC11812_A	F	F	-	L	L	T	G	I	V
	OsFPS2_A	Y	F	-	L	L	T	G	I	V
	At AAL34286_B	Y	F	-	L	L	T	G	I	V
	At CAB80990_B	Y	F	-	L	L	T	G	I	V
	Nt AHM22931_B	Y	F	-	L	L	T	G	I	V
	Sm ABV08819_B	Y	F	-	L	L	T	G	I	V

Table S6. Spatial and temporal expression of rice *trans* -PT genes.

	Seedling stage		Vegitative stage				Reproductive stage			Seed
**Gene Names**	**L**	**R**	**6L**	**6R**	**9L**	**9R**	**L**	**R**	**F**	***Se***
OsGPS	0.003 ± 0.001	0.008 ± 0.004	0.001 ± 0.000	0.001 ± 0.000	0.012 ± 0.001	0.001 ± 0.000	0.029 ± 0.004	0.002 ± 0.000	0.003 ± 0.001	0.006 ± 0.001
OsGGPS1	0.050 ± 0.005	0.007 ± 0.000	0.102 ± 0.002	0.018 ± 0.001	0.019 ± 0.001	0.003 ± 0.001	0.019 ± 0.001	0.006 ± 0.001	0.007 ± 0.001	0.009 ± 0.001
OsGRP	0.020 ± 0.002	0.010 ± 0.001	0.176 ± 0.007	0.022 ± 0.002	0.028 ± 0.001	0.004 ± 0.000	0.03 ± 0.002	0.009 ± 0.002	0.01 ± 0.003	0.019 ± 0.003
OsSPS1	0.013 ± 0.000	0.010 ± 0.001	0.02 ± 0.001	0.017 ± 0.000	0.006 ± 0.001	0.005 ± 0.001	0.005 ± 0.000	0.006 ± 0.000	0.004 ± 0.000	0.013 ± 0.001
OsSPS2	0.093 ± 0.003	0.013 ± 0.000	0.021 ± 0.001	0.007 ± 0.000	0.481 ± 0.010	0.003 ± 0.000	0.116 ± 0.001	0.01 ± 0.001	0.043 ± 0.002	0.029 ± 0.001
OsSPS3	0.007 ± 0.001	0.004 ± 0.000	0.011 ± 0.000	0.037 ± 0.002	0.012 ± 0.001	0.002 ± 0.000	0.01 ± 0.001	0.004 ± 0.000	0.005 ± 0.000	0.015 ± 0.001
OsSPS4	0.044 ± 0.005	0.006 ± 0.001	0.025 ± 0.004	0.000 ± 0.000	0.07 ± 0.004	0.000 ± 0.000	0.03 ± 0.001	0.003 ± 0.000	0.005 ± 0.000	0.011 ± 0.002
OsFPS1	0.235 ± 0.009	0.039 ± 0.004	0.074 ± 0.001	0.032 ± 0.001	0.602 ± 0.031	0.026 ± 0.000	0.594 ± 0.071	0.033 ± 0.005	0.151 ± 0.02	0.074 ± 0.007
OsFPS2	0.054 ± 0.006	0.107 ± 0.007	0.016 ± 0.000	0.204 ± 0.011	0.014 ± 0.001	0.126 ± 0.003	0.015 ± 0.001	0.082 ± 0.001	0.048 ± 0.003	0.024 ± 0.002
OsFPS3	0.021 ± 0.002	0.013 ± 0.001	0.06 ± 0.002	0.017 ± 0.000	0.322 ± 0.011	0.005 ± 0.000	0.228 ± 0.008	0.007 ± 0.001	0.060 ± 0.007	0.073 ± 0.010
OsFPS4	0.033 ± 0.002	0.000 ± 0.000	0.032 ± 0.000	0.000 ± 0.000	0.149 ± 0.005	0.000 ± 0.000	0.054 ± 0.001	0.000 ± 0.000	0.003 ± 0.000	0.000 ± 0.000

All data are expressed as the mean values ± standard error from three technical replicates. The expression levels of the above genes are presented as ΔCq values. L, leaves; R, roots; F, florets; Se, seeds harbested at 40 days after floweing.

Table S7. Expression profiles of rice *trans* -PT genes in MeJA, ABA and GA-treated rice leaves.

†Chemicals	Gene Names	¶ΔCq ± SE	Fold change ($2^{-\Delta\Delta Ct}$) ± SE					
		0	0.5	1	2	4	6	(hpt)
**Mock**	*OsGPS*	0.004 ± 0.000	0.990 ± 0.460	0.779 ± 0.203	1.264 ± 0.728	1.223 ± 0.169	0.858 ± 0.168	
	OsGGPS1	0.004 ± 0.000	0.754 ± 0.141	1.047 ± 0.046	1.281 ± 0.069	0.915 ± 0.036	0.845 ± 0.087	
	OsGRP	0.036 ± 0.001	0.767 ± 0.080	0.803 ± 0.059	0.974 ± 0.091	0.681 ± 0.023	0.728 ± 0.093	
	OsSPS1	0.011 ± 0.001	0.91 ± 0.068	0.909 ± 0.058	0.983 ± 0.034	0.926 ± 0.038	0.910 ± 0.010	
	OsSPS2	0.082 ± 0.011	0.877 ± 0.014	1.072 ± 0.091	0.911 ± 0.109	1.079 ± 0.088	0.739 ± 0.049	
	OsSPS3	0.043 ± 0.002	0.851 ± 0.022	1.026 ± 0.133	1.354 ± 0.206	1.012 ± 0.071	0.817 ± 0.027	
	OsSPS4	0.024 ± 0.001	0.875 ± 0.059	0.959 ± 0.078	0.953 ± 0.054	1.24 ± 0.059	0.919 ± 0.103	
	OsFPS1	0.448 ± 0.006	0.959 ± 0.140	0.931 ± 0.074	0.745 ± 0.030	0.845 ± 0.016	0.744 ± 0.138	
	OsFPS2	0.196 ± 0.008	0.787 ± 0.025	0.793 ± 0.100	0.780 ± 0.025	0.869 ± 0.061	0.831 ± 0.099	
	OsFPS3	0.090 ± 0.010	0.950 ± 0.041	0.946 ± 0.015	0.783 ± 0.055	0.979 ± 0.078	0.642 ± 0.011	
	OsFPS4	0.085 ± 0.010	0.862 ± 0.070	0.883 ± 0.037	0.688 ± 0.012	0.948 ± 0.079	0.667 ± 0.007	
**MeJA**	*OsGPS*	0.002 ± 0.000	2.425 ± 0.358	10.715 ± 1.520	30.635 ± 6.379	6.070 ± 0.294	7.634 ± 2.851	
	OsGGPS1	0.002 ± 0.000	0.996 ± 0.016	1.876 ± 0.053	0.815 ± 0.025	2.558 ± 0.093	2.156 ± 0.197	
	OsGRP	0.025 ± 0.003	0.900 ± 0.121	0.749 ± 0.039	0.831 ± 0.055	0.712 ± 0.086	0.624 ± 0.039	
	OsSPS1	0.004 ± 0.000	0.774 ± 0.000	1.885 ± 0.190	1.668 ± 0.047	1.603 ± 0.029	0.999 ± 0.055	
	OsSPS2	0.076 ± 0.002	1.297 ± 0.046	1.176 ± 0.146	0.605 ± 0.032	0.651 ± 0.073	0.601 ± 0.069	
	OsSPS3	0.037 ± 0.005	1.283 ± 0.138	0.786 ± 0.123	0.401 ± 0.083	0.447 ± 0.050	0.460 ± 0.012	
	OsSPS4	0.022 ± 0.001	1.060 ± 0.076	0.741 ± 0.009	0.713 ± 0.027	0.77 ± 0.083	0.618 ± 0.042	
	OsFPS1	0.218 ± 0.010	1.204 ± 0.086	2.713 ± 0.129	2.086 ± 0.050	1.655 ± 0.032	1.166 ± 0.104	
	OsFPS2	0.104 ± 0.013	0.712 ± 0.060	1.118 ± 0.016	0.961 ± 0.073	0.768 ± 0.407	0.731 ± 0.104	
	OsFPS3	0.032 ± 0.001	1.146 ± 0.263	2.316 ± 0.235	0.870 ± 0.145	0.507 ± 0.070	0.818 ± 0.045	
	OsFPS4	0.027 ± 0.004	0.983 ± 0.060	0.424 ± 0.150	0.129 ± 0.03	0.196 ± 0.020	0.164 ± 0.031	
**ABA**	*OsGPS*	0.000 ± 0.000	0.814 ± 0.054	0.730 ± 0.086	0.422 ± 0.019	0.295 ± 0.050	0.771 ± 0.369	
	OsGGPS1	0.004 ± 0.000	1.108 ± 0.111	0.833 ± 0.131	1.775 ± 0.270	1.279 ± 0.092	0.924 ± 0.073	
	OsGRP	0.035 ± 0.003	0.596 ± 0.037	0.572 ± 0.015	0.937 ± 0.114	0.833 ± 0.038	0.603 ± 0.010	
	OsSPS1	0.006 ± 0.000	1.193 ± 0.446	0.690 ± 0.070	1.290 ± 0.143	1.231 ± 0.152	0.650 ± 0.331	
	OsSPS2	0.102 ± 0.004	0.969 ± 0.028	0.927 ± 0.043	1.748 ± 0.135	2.528 ± 0.282	1.755 ± 0.161	
	OsSPS3	0.034 ± 0.002	0.753 ± 0.042	0.838 ± 0.078	1.136 ± 0.160	1.042 ± 0.069	0.631 ± 0.130	
	OsSPS4	0.015 ± 0.001	0.579 ± 0.197	0.580 ± 0.100	1.299 ± 0.180	0.789 ± 0.021	0.571 ± 0.286	
	OsFPS1	0.388 ± 0.023	0.547 ± 0.049	0.578 ± 0.018	1.234 ± 0.059	1.389 ± 0.074	0.751 ± 0.041	
	OsFPS2	0.141 ± 0.014	0.849 ± 0.072	0.759 ± 0.062	0.922 ± 0.036	0.689 ± 0.024	0.582 ± 0.052	
	OsFPS3	0.043 ± 0.005	0.801 ± 0.051	0.781 ± 0.054	2.052 ± 0.191	2.435 ± 0.049	1.702 ± 0.233	
	OsFPS4	0.034 ± 0.004	1.039 ± 0.064	0.888 ± 0.093	1.675 ± 0.093	1.561 ± 0.069	0.968 ± 0.041	
**GA**	*OsGPS*	0.001 ± 0.000	0.832 ± 0.224	0.601 ± 0.170	0.355 ± 0.134	0.247 ± 0.076	0.603 ± 0.190	
	OsGGPS1	0.026 ± 0.001	1.153 ± 0.044	1.287 ± 0.093	0.955 ± 0.046	1.033 ± 0.166	0.903 ± 0.043	
	OsGRP	0.018 ± 0.001	1.307 ± 0.083	1.308 ± 0.148	1.059 ± 0.102	1.195 ± 0.097	1.013 ± 0.109	
	OsSPS1	0.007 ± 0.000	1.326 ± 0.024	1.554 ± 0.069	1.171 ± 0.03	1.413 ± 0.18	1.073 ± 0.066	
	OsSPS2	0.149 ± 0.013	1.075 ± 0.058	1.093 ± 0.032	0.801 ± 0.068	0.929 ± 0.103	0.685 ± 0.035	
	OsSPS3	0.050 ± 0.003	0.684 ± 0.028	0.693 ± 0.008	0.591 ± 0.056	0.669 ± 0.044	0.538 ± 0.031	
	OsSPS4	0.013 ± 0.000	1.021 ± 0.069	0.808 ± 0.056	0.688 ± 0.076	0.562 ± 0.023	0.521 ± 0.042	
	OsFPS1	0.209 ± 0.026	1.354 ± 0.094	1.118 ± 0.084	1.167 ± 0.126	0.765 ± 0.009	0.991 ± 0.151	
	OsFPS2	0.101 ± 0.001	0.939 ± 0.064	1.117 ± 0.075	1.044 ± 0.081	1.165 ± 0.060	1.106 ± 0.342	
	OsFPS3	0.023 ± 0.001	1.128 ± 0.011	1.040 ± 0.087	1.014 ± 0.087	0.641 ± 0.037	0.770 ± 0.072	
	OsFPS4	0.023 ± 0.001	1.374 ± 0.104	1.200 ± 0.094	1.174 ± 0.220	0.874 ± 0.034	1.016 ± 0.102	

All data are expressed as the mean values ± standard error from three technical replicates. The expression levels of the above genes are presented as ΔCq values for 0 hour-post chemical treatment (hpt), and as the fold change values ($2^{-\Delta\Delta Ct}$) relative to it for other time-series samples (¶). ABA, 100 µM absisic acid; GA, 100 µM giberellic acid; MeJA,100 µM methyl jasmonic acid; Mock, treated only with buffer.

Figure S1

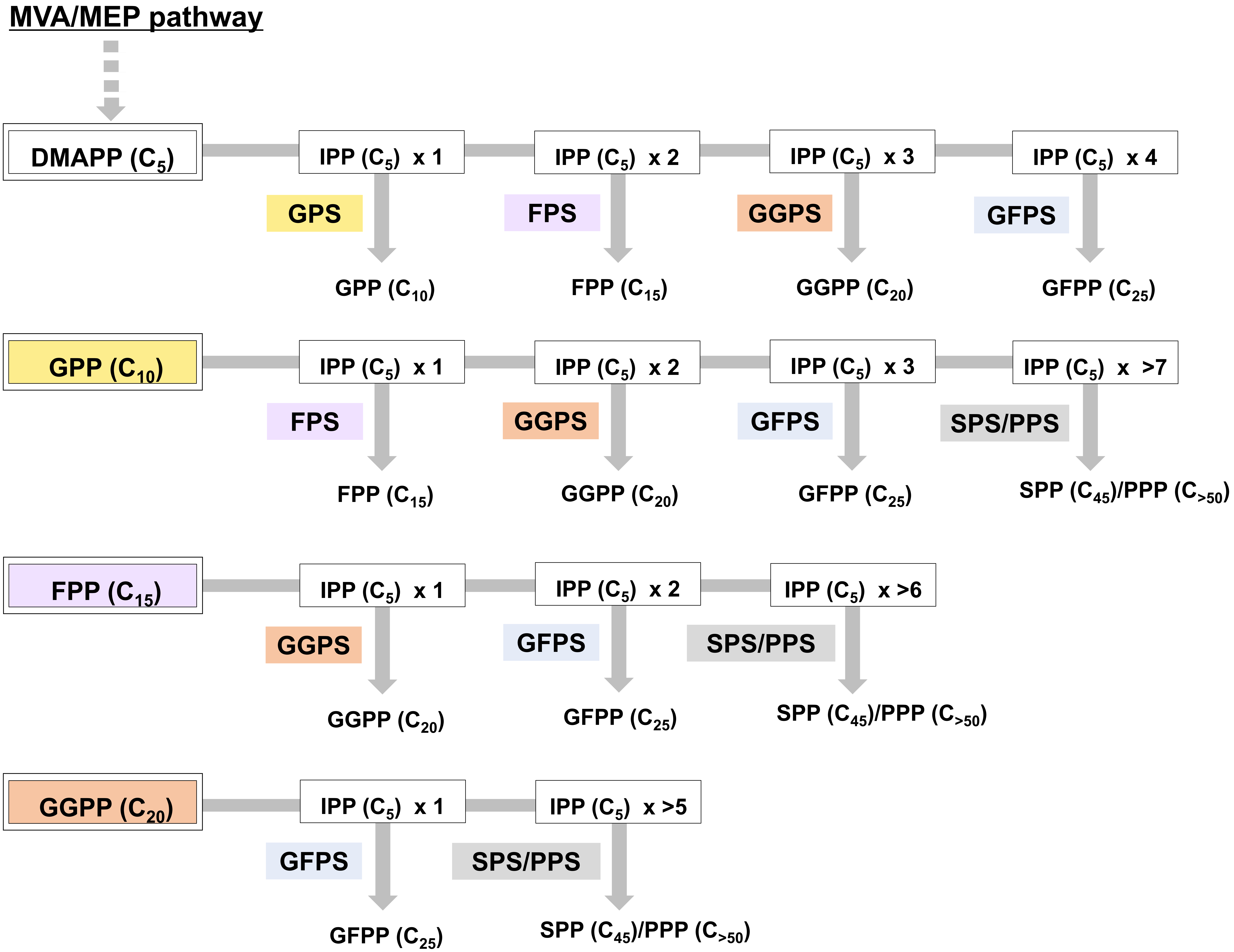

MVA/MEP pathway
DMAPP (C_5)
IPP (C_5) x 1
IPP (C_5) x 2
IPP (C_5) x 3
IPP (C_5) x 4
GPS
FPS
GGPS
GFPS
GPP (C_{10})
FPP (C_{15})
GGPP (C_{20})
GFPP (C_{25})
GPP (C_{10})
IPP (C_5) x 1
IPP (C_5) x 2
IPP (C_5) x 3
IPP (C_5) x >7
FPS
GGPS
GFPS
SPS/PPS
FPP (C_{15})
GGPP (C_{20})
GFPP (C_{25})
SPP (C_{45})/PPP ($C_{>50}$)
FPP (C_{15})
IPP (C_5) x 1
IPP (C_5) x 2
IPP (C_5) x >6
GGPS
GFPS
SPS/PPS
GGPP (C_{20})
GFPP (C_{25})
SPP (C_{45})/PPP ($C_{>50}$)
GGPP (C_{20})
IPP (C_5) x 1
IPP (C_5) x >5
GFPS
SPS/PPS
GFPP (C_{25})
SPP (C_{45})/PPP ($C_{>50}$)

Figure S2

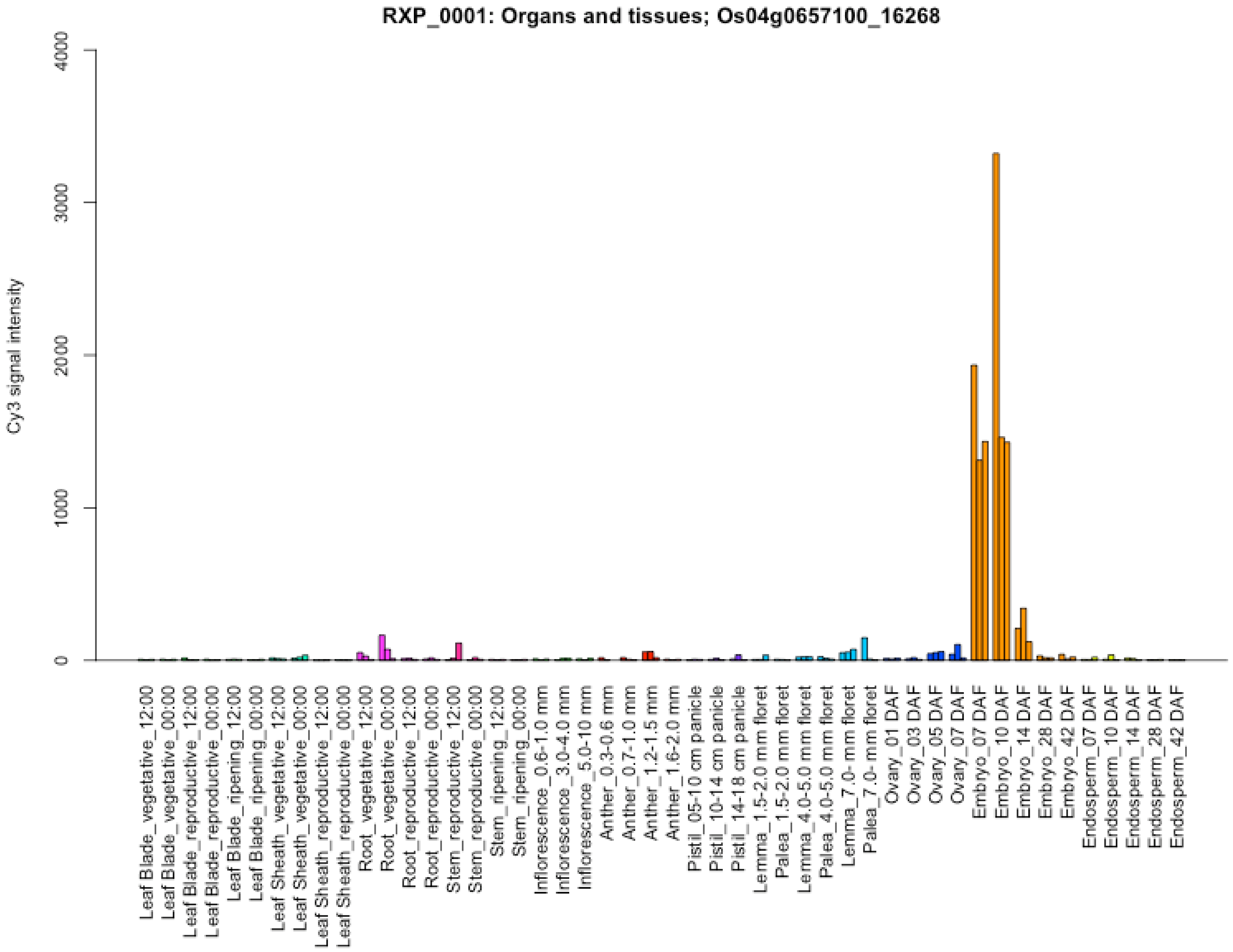

RXP_0001: Organs and tissues; Os04g0657100_16268
Cy3 signal intensity
0
1000
2000
3000
4000
Leaf Blade_vegetative_12:00
Leaf Blade_vegetative_00:00
Leaf Blade_reproductive_12:00
Leaf Blade_reproductive_00:00
Leaf Blade_ripening_12:00
Leaf Blade_ripening_00:00
Leaf Sheath_vegetative_12:00
Leaf Sheath_vegetative_00:00
Leaf Sheath_reproductive_12:00
Leaf Sheath_reproductive_00:00
Root_vegetative_12:00
Root_vegetative_00:00
Root_reproductive_12:00
Root_reproductive_00:00
Stem_reproductive_12:00
Stem_reproductive_00:00
Stem_ripening_12:00
Stem_ripening_00:00
Inflorescence_0.6-1.0 mm
Inflorescence_3.0-4.0 mm
Inflorescence_5.0-10 mm
Anther_0.3-0.6 mm
Anther_0.7-1.0 mm
Anther_1.2-1.5 mm
Anther_1.6-2.0 mm
Pistil_05-10 cm panicle
Pistil_10-14 cm panicle
Pistil_14-18 cm panicle
Lemma_1.5-2.0 mm floret
Palea_1.5-2.0 mm floret
Lemma_4.0-5.0 mm floret
Palea_4.0-5.0 mm floret
Lemma_7.0- mm floret
Palea_7.0- mm floret
Ovary_01 DAF
Ovary_03 DAF
Ovary_05 DAF
Ovary_07 DAF
Embryo_07 DAF
Embryo_10 DAF
Embryo_14 DAF
Embryo_28 DAF
Embryo_42 DAF
Endosperm_07 DAF
Endosperm_10 DAF
Endosperm_14 DAF
Endosperm_28 DAF
Endosperm_42 DAF

Figure S3

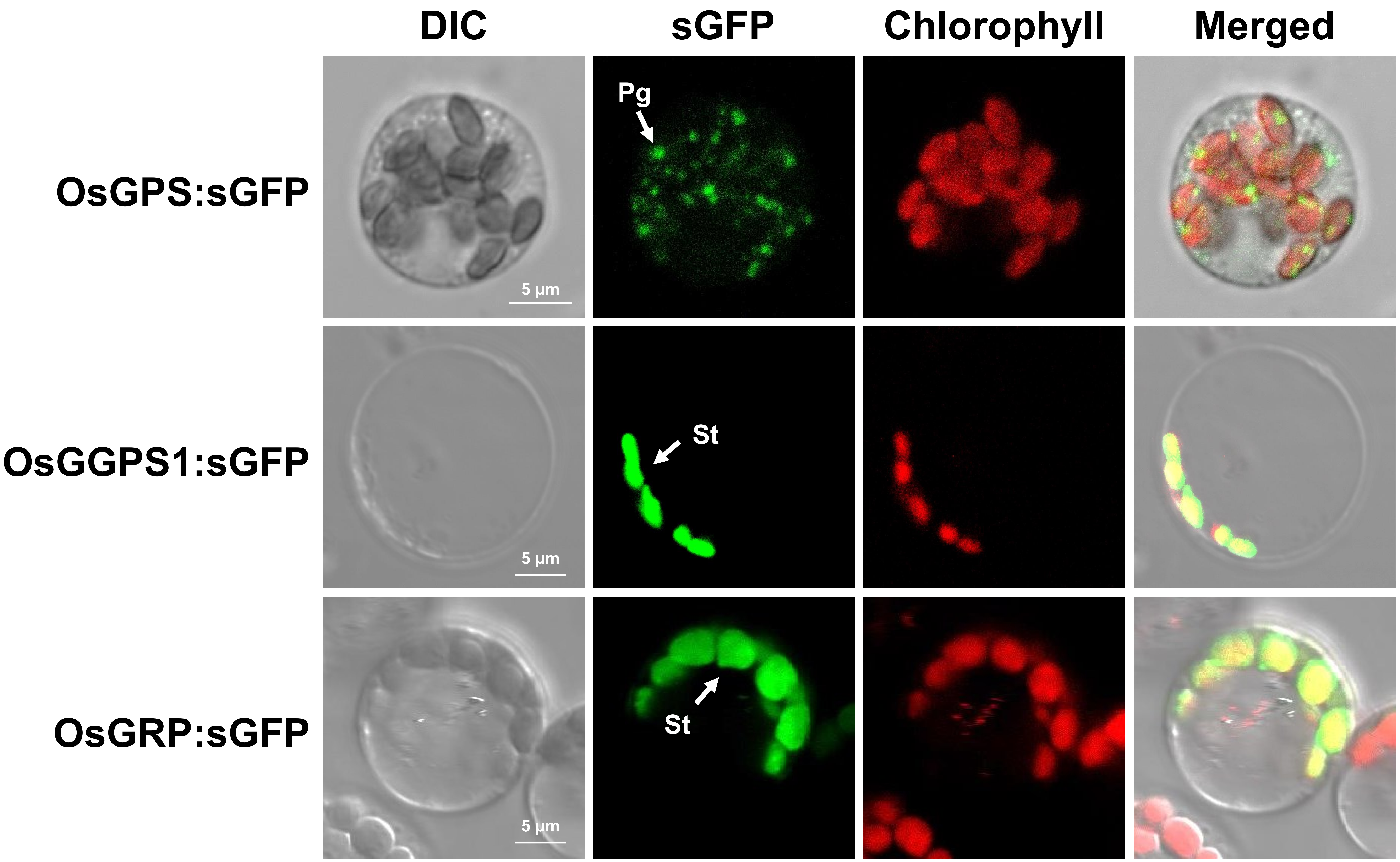
DIC
sGFP
Chlorophyll
Merged
OsGPS:sGFP
OsGGPS1:sGFP
OsGRP:sGFP
Pg
St
St
5 μm
5 μm
5 μm
